# Editorial: Activities of daily living and everyday functioning: From normal aging to neurodegenerative diseases

**DOI:** 10.3389/fnagi.2023.1161736

**Published:** 2023-03-20

**Authors:** Ondrej Bezdicek, Joaquim Ferreira, Robert Fellows, Inga Liepelt-Scarfone

**Affiliations:** ^1^Department of Neurology and Centre of Clinical Neuroscience, First Faculty of Medicine and General University Hospital in Prague, Charles University, Prague, Czechia; ^2^Laboratory of Clinical Pharmacology and Therapeutics, Faculdade de Medicina, Universidade de Lisboa, Lisbon, Portugal; ^3^Department of Psychiatry, University of California, San Diego, San Diego, CA, United States; ^4^German Center for Neurodegenerative Diseases (DZNE) and Hertie Institute for Clinical Brain Research, Eberhard Karls University Tübingen, Tübingen, Germany; ^5^IB-Hochschule, Stuttgart, Germany

**Keywords:** activities of daily living, cognition, mild cognitive impairment, neurodegeneration, functional ability

Activities of daily living (ADLs) are a wide-ranging psychological construct to designate and measure individuals' independence and range of everyday functioning in a social environment. Populations in developed countries are aging and estimates of those over the age of 85 who will need assistance with ADLs have tripled. An even steeper decline in ADLs can be expected in people at risk of developing neurodegenerative or other types of diseases (Buchman et al., 2009; Gill et al., 2013). Deficits in ADLs due to cognitive impairment are increasingly discussed as a risk factor for dementia in older age and neurodegenerative diseases (Verlinden et al., 2016; Becker et al., 2022; Jang et al., 2022; Ng et al., 2022), indicating a need for sensitive assessments, especially in mild stages of ADL difficulty. ADLs are affected by multiple factors such as physical fitness, cognitive ability, and mental health (Cahn et al., 1998; Tam et al., 2008; Christensen et al., 2013). Thus, basic and applied research on ADLs is highly necessary as any psychological, medical, or technological intervention that helps to promote or preserve ADLs in later adulthood is of the utmost importance to preserve social independence and goes far beyond this: it helps to diminish the caregiver burden in each family, it decreases healthcare costs, and it creates an existential perspective that life is worth living.

Thus, the current *Research Topic on Activities of Daily Living and Everyday Functioning: From Normal Aging to Neurodegenerative Diseases* is dedicated to different areas of basic and applied research.

Gait disturbances and falls constitute a major reason for disability in older age as fall incidence can result in fractures and other injuries (Lord and Close, 2018). Cerebral small vessel disease (CSVD) has been related to gait disturbances (Markus and De Leeuw, 2023); however, the role of enlarged perivascular spaces in the basal ganglia on these disabilities is still in debate. Data from Yang S. et al. identified that a greater number of enlarged perivascular spaces in the basal ganglia were independently related to gait disturbances in older people with CSVD. Fear of falling (FoF) is associated with poorer physical and cognitive functions and decreasing ability to perform ADLs; it has been identified as a potential risk factor for falls in older age and neurodegenerative diseases (Bryant et al., 2013; Schoene et al., 2019). The article by Atrsaei et al. evaluates the influence of FoF on mobility in patients with Parkinson's disease (PD), highlighting the importance of monitoring different environments and assessment strategies. In a meta-analysis, Kim et al. found that task-specific reactive balance exercise training may be an optimal intervention in preserving reactive balance to prevent falls in older age. Taken together, these studies identify brain morphological and multimodal clinical risk factors for decreased mobility, as well as targeted strategies for reducing falls and improving functioning. All contributions underline the importance of vascular, psychological, and prophylactic factors in falls and in their monitoring for a better prediction of treatment strategies.

PD is a multicomplex neurodegenerative disease comprising both motor and non-motor symptoms affecting ADLs. For the identification of ADLs impairment indicative of dementia in PD, valid ratings of motor and cognitive sources primarily affecting ADLs are essential (Dubois et al., 2007). The article by Becker et al. focuses on the agreement between self- and informant-reported ADLs and their association with cognitive performance in patients with PD. Of note, motor severity showed a high impact on both self- and informant-reported functioning. Their research indicates a need for objective ADLs measures because the agreement of patient and informant ratings of ADLs function showed only moderate agreement. The contribution of Rehman et al. applies modern machine learning methods to the analysis of gait in PD and controls in real-world and laboratory characteristics. Another line of research in PD is dedicated to non-motor effects and ADLs in patients undergoing deep brain stimulation of the subthalamic nucleus. The study by Bezdicek et al. shows a positive effect of the treatment on instrumental ADLs in the post-surgery phase. In sum, these contributions highlight novel approaches to evaluate the validity of assessments and monitor the effects of treatment on ADLs in neurodegenerative diseases such as PD.

Psychiatric diseases are known to affect patients' behavior and everyday life. Depression is common in older age, especially in patients with mild cognitive impairment (MCI; Ismail et al., 2017). The article by Numbers et al. revealed that patients' self- and caregiver ratings of ADLs are associated with the severity of depression in a community-dwelling older group. In contrast, objective measures seem to be more robust against the influence of depression and personality and might therefore be a suitable alternative to differentiate between cognitive and psychiatric effects on ADLs disabilities. Similarly, Yang D. et al. show that a certain time and proportion per week of vigorous to moderate physical activity in men over 45 years of age lowers the risk of depression. These studies emphasize the importance of preventive measures in preserving ADLs in relation to neuropsychiatric symptoms such as depression.

In addition to the identification of risk and modulating factors of ADLs in older age and neurodegenerative diseases, treatment strategies are essential to prevent the progression of ADLs impairment. In their systematic review and meta-analysis, Han et al. concluded that combined cognitive and physical intervention enhances cognition in older adults in the short term irrespective of patients' cognitive status. However, to get insight into long-term treatment effects in older adults, additional high-quality studies are needed. The effects of multimodal exercise on health outcomes in community-dwelling older adults are the focus of the article of Vogel et al.. Their data support the assumption that the training outcome depends on factors like sleep duration, movement biography, and activity profile. Therefore, the identification of factors maximizing the treatment outcome of specific therapies is crucial for patients' differential treatment indication.

To date, the pathological mechanisms leading to ADLs impairment are only partly understood. The work of Fellows et al. concluded that larger white matter hyperintensity and smaller hippocampal volumes are correlates for poorer everyday function. They also identified unique contributions of cognitive measures to a newly developed index of pathological functional impairment and neuropsychiatric symptoms to functional reserve in MCI and healthy older adults. Frailty is associated with lower health-related quality of life (Solfrizzi et al., 2019; Chen et al., 2022) and increases the risk of disability, dementia, and mortality (Yi and Yoon, 2023). Most interestingly, abnormalities in the integrity of the left anterior thalamic radiation seem to be associated with frailty in patients with cardiometabolic diseases as shown by Tamura et al.. Also, the prevalence of MCI, which is a risk factor for lower ADLs functioning (Perneczk et al., 2006), according to the study of Liu et al., is higher in women than in men of older age. This group of studies uncovers the pathological and demographic factors at play in the development of ADLs impairment.

In conclusion, the Research Topic gives an overview of the state-of-the-art research on ADLs in healthy aging and different clinical conditions. It highlights the key areas of contemporary research into ADLs so that individuals can participate in social life events even at an older age.

## Author contributions

All authors listed have made a substantial, direct, and intellectual contribution to the work and approved it for publication.
